# Cobalt Cardiomyopathy Secondary to Hip Arthroplasty: An Increasingly Prevalent Problem

**DOI:** 10.1155/2017/5434571

**Published:** 2017-08-06

**Authors:** Russel Tilney, Melanie Roberta Burg, Mark Adrian Sammut

**Affiliations:** ^1^Department of Medicine, Mater Dei Hospital, Msida, Malta; ^2^Department of Cardiology, Mater Dei Hospital, Msida, Malta

## Abstract

A forty-year-old man experienced worsening heart failure four years following bilateral complicated total hip replacement. His condition was extensively worked up but no underlying pathology was immediately evident. Given the cobalt-chromium alloy component present in the hip arthroplasties, the raised cobalt blood levels, and a fitting clinical picture coupled with radiological findings, the patient underwent right hip revision. Evidence of biotribocorrosion was present on direct visualisation intraoperatively. The patient subsequently experienced symptomatic improvement (NYHA class III to class I) and echocardiography showed recovery of ejection fraction. Cobalt exists as a bivalent and trivalent molecule in circulation and produces a cytotoxicity profile similar to nanoparticles, causing neurological, thyroid, and cardiological pathology. Blood levels are not entirely useful as there is no identifiable conversion factor for levels in whole blood, serum, and erythrocytes which seem to act independently of each other. Interestingly cobalt cardiomyopathy is frequently compounded by other possible causes of cardiomyopathy such as alcohol and a link has been postulated. Definitive treatment is revision of the arthroplasty as other treatments are unproven.

## 1. Introduction

Systemic cobalt toxicity has been linked to neurological, cardiological, and endocrinological pathology. As of January 2016 there are only 18 cases where cobalt toxicity has been attributed to cobalt-chromium alloys in hip arthroplasties; however causality has not been established. Approximately one million arthroplasties worldwide have been performed with cobalt components, mostly between 2003 and 2010.

This case report discusses the possibility of metal-on-metal biotribocorrosion leading to severe cardiomyopathy with partial symptomatic and echocardiographic resolution on revision of the hip arthroplasty in a 40-year-old gentleman.

## 2. Case Presentation

A forty-year-old man presented with a two-month history of exertional dyspnoea and intermittent palpitations. The symptoms had worsened significantly in the few days preceding admission and he complained of orthopnoea and a persistent dry cough. He denied any antecedent infections but admitted to occasional cocaine and alcohol use. Examination at this time was unremarkable except for a persistent tachycardia of 126 beats per minute. Electrocardiography confirmed sinus tachycardia. Chest X-ray showed an increased cardiothoracic ratio and a globular heart.

His past history was significant for bilateral total hip replacement 4 years prior to presentation for a 2-year history of hip pain and inability to weight bear. Avascular necrosis was suspected. The procedure on the right was complicated by loosening of the acetabular cap which required reoperation one day postoperatively. The DePuy Corail® Total Hip System (cementless femoral stem without collar) was used bilaterally.

A bedside echocardiogram during this admission revealed severely impaired left ventricular systolic function in the absence of gross dilatation. The left ventricle also appeared hypertrophied. In addition, a circumferential pericardial effusion of significant size was noted. Ejection fraction was estimated at 40–45% by Simpson's biplane method on echocardiography.

Pericardiocentesis was carried out: a total of 1.32 L of straw-coloured clear pericardial fluid was aspirated and sent for testing. Bacterial culture (including* Mycobacterium tuberculosis *PCR) and cytology were negative. Biochemistry was compatible with a transudate.

He underwent extensive workup for possible causes: EBV; CMV; HEP screen (HBsAg, HAV IgM, and anti-HCV antibody);* Rickettsia conorii* IgM,* M. pneumoniae*, ANA, total ENA, RF, ANCA, total IgM, IgA, and IgG, all tested negative.

Following pericardiocentesis the cardiac contractility was observed to improve significantly on serial echocardiography; however ejection fraction did not improve. Speckling of the myocardium was, however, noted. Endomyocardial biopsy was performed which revealed no evidence of amyloid deposition on Congo red staining. Mild perivascular fibrosis around a few intramural vessels and mild vacuolation of a few cardiomyocytes was seen.

A repeat bedside echocardiogram showed reaccumulation of the pericardial effusion and the patient was subsequently started on colchicine 500 micrograms BD. His antifailure treatment was optimized. The pericardial effusion continued to recur, so definitive management of the pericardial effusion with a pericardial window was performed.

The patient was referred back to hospital from primary healthcare in view of a vague epigastric discomfort together with tachycardia. ECG confirmed new onset atrial flutter at 176 beats/min. He was anticoagulated with heparin and warfarin and urgent DC electrocardioversion was performed at 100 J under general anaesthetic, restoring sinus rhythm without any complications.

On this occasion, echocardiography showed severely impaired biventricular function with an LVEF 20% using Simpson's biplane method and advanced diastolic dysfunction with restrictive transmitral filling and raised filling pressure and a small localised pericardial effusion (inferolateral/inferior aspect of LV).

Right and left heart studies showed normal coronary arteries. Cardiac output was estimated at 3.5 L/min by temperature haemodilution method.

Given the past history of bilateral hip arthroplasty and persistent right-sided hip pain, the possibility of cardiomyopathy secondary to cobalt heavy metal poisoning due to a metal-on-metal (MoM) reaction from the hip prostheses was discussed. Whole blood cobalt levels were 4174 nmol/L (reference range < 5 nmol/L). The case was brought to the attention of the orthopaedic surgeon who performed the hip replacements and who confirmed that a cobalt-chromium alloy component was present (reference numbers provided below).

Ultrasound imaging of the right hip demonstrated an inhomogenous, generally hypoechoic fluid collection with internal scattered echogenic debris measuring 8 cm × 3 cm × 2.5 cm located between the skin and the right greater trochanter. The reporting radiologist suggested that this may represent an aseptic lymphocyte-dominated vasculitic-associated lesion reaction.

The left hip appeared largely unremarkable.

The need for right hip revision was discussed with the patient who understood and agreed.

Eight months later, revision of right total hip replacement was performed. Blackening of the gluteal muscle and greater trochanter secondary to metallosis was noted. The femoral stem was solid; however the head was dislocated. Thorough capsulotomy and synovectomy were performed. Postoperatively he was managed on ITU in view of his cardiomyopathy. Histology demonstrated reparative and inflammatory changes with metallosis.

Five months later, he had a much better exercise tolerance (NYHA class III improved to class I) and estimated left ventricular ejection fraction on echocardiography improved to 45–50%.

## 3. Discussion

Cobalt is a trace metal element that is essential for the production of cobalamin permitting normal cellular function; however, in excess levels it may cause cellular damage: apoptosis, necrosis, and oxidative damage to DNA [1]. Cobalt toxicity first became evident following use of cobalt-chloride as a haematopoietic agent in anaemic individuals when it was noted to be goitrogenic [2, 3] and neurotoxic [3]. Low-output cardiomyopathy was identified in individuals who drank excessive amounts of “Brand XXX” beer in Quebec between 1962 and 1965; the beer was later found to have significantly higher levels of cobalt which was used as a foam-stabilising agent [4] and the term “Beer-drinker's Cardiomyopathy” was coined.

Over the last 15 years there has been an increase in the use of MoM total hip arthroplasties (THAs) and fears that soft tissue reactions may be on the increase have prompted the Medical and Healthcare products Regulatory Agency (MHRA) to issue a medical device alert for these prostheses [5]. Monitoring of cobalt and chromium levels in these patients is now recommended [5].

The process through which cobalt becomes available to interact with human physiology is termed biotribocorrosion (tribocorrosion within a biological environment). The tribological aspect describes the mechanical behaviour causing wear and tear, whilst corrosion refers to the electrochemical component of the process. Cobalt, once released into the systemic circulation, exists as a bivalent or trivalent nanoparticle and has a cytotoxicity profile similar to other nanoparticles (producing neuronal, cardiomyocyte, and thyroid damage through apoptosis, necrosis, and DNA damage) [1]. Numerous cobalt-containing nanoparticles have been identified through hip MoM simulations; however the exact biochemical mechanism through which they are produced remains unclear [6].

For THAs, tribological damage typically occurs through wear and tear of two surfaces that are intended to articulate with each other, through third-body wear (such as particles within the joint space), and through non-weight-bearing surfaces [7] (such as micromotion at the modular components of prostheses) [8].

Our patient presented with typical symptoms of cardiac cobalt toxicity, that is, dyspnoea and symptoms on exertion stemming from a low-output dilated cardiomyopathy (although our patient did not have significant dilatation). Atrial fibrillation and flutter and pericardial effusion have also been linked [1]. The pathophysiology is ill-defined but decreased oxygen uptake resulting in increased intracellular cardiomyocyte calcium levels and sympathetic tone has been suggested [9].

**Figure 1:**
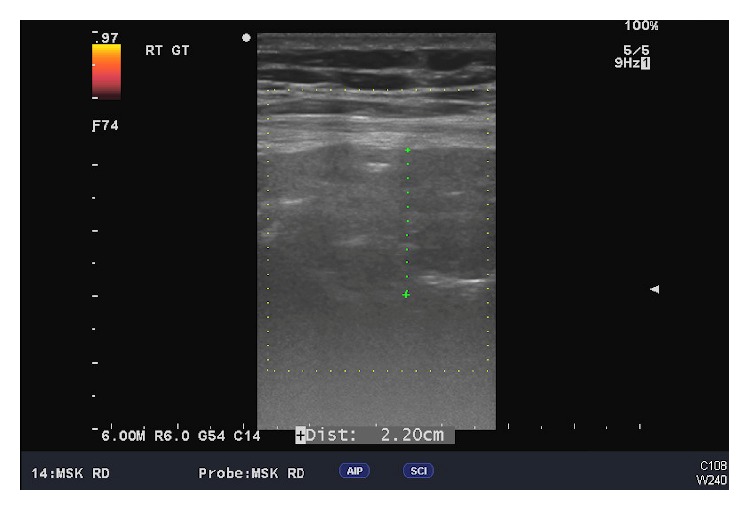
Ultrasound of the right hip showing an inhomogeneous hypoechoic fluid collection with internally scattered echogenic debris between the skin and the right greater trochanter measuring approximately 8 × 3 × 2.5 cm which raised the suspicion of a metal-on-metal ALVAL reaction.

Ultrasound of our patient's hips (Figure 1()) revealed a cystic mass associated with the right hip THA which is classically described as a “pseudotumour,” a noninfectious, nonneoplastic mass [10] which results from an ALVAL that produces a type IV hypersensitivity reaction [11]. The clinical features of this underrecognised condition are not specific but pain is typical. Findings suggestive of chronic inflammation related to ALVAL intraoperatively mandate revision of the articular surfaces. Dense perivascular inflammatory infiltrates are characteristic of ALVAL [11].

Cobalt levels are not useful in defining toxicity and thresholds vary substantially from one study to the next [12]. In addition, levels may be measured from serum, whole blood, or erythrocytes [13] and there is no conversion factor for these values as the intracellular and extracellular compartments act independently of each other [14]. Cobalt and chromium levels, or cobalt levels alone, may be used to identify patients at risk of adverse reactions to metal debris [15].

At this point a link between cobalt toxicity and systemic effects in the literature has not been shown. Furthermore, in this case report it is important to recognise that other causes may be present in this case. Both alcohol and cocaine may cause dilated cardiomyopathy [13, 16]. Alternative diagnoses must be considered especially since thyroid dysfunction and neurological deficits were absent. It is intriguing to note, however, that other cases were also confounded by alternative possible causes such as alcohol and it has been postulated that this may have compounded the clinical findings [17].

Treatment with chelating agents has been attempted but has not been rigorously tested [18]. Addition of amino acids has also been tried in order to increase cellular transport of the chelators [18]. Haemodialysis is suspected to not have a profound effect on cobalt levels because it is primarily protein bound [19]. The definitive treatment for cobalt toxicity in the setting of implant related causes is therefore surgical removal of the implant [20].

In conclusion, clinical vigilance is needed to diagnose and manage cobalt toxicity in patients who have undergone THAs in the past. At present the number of cases is small but, with the large number of MoM THAs that have been performed in the past, the likelihood is that we shall be seeing more of these cases in the near future.

## Supplementary Material

Transthoracic echo prior to hip revision (apical 4 chamber [A4C], parasternal long axis [PSLAX] and parasternal short axis [PSSAX] views - September 2014), and post hip revision (October 2015) illustrating clear improvement in left ventricular systolic function.

## Data Access

DePuy THA reference numbers used in our patient are as follows:* left hip*: acetabular implant, 9998-04-754; taper sleeve adaptor, 9998-00-108; femoral implant, 9998-90-147; femoral stem, 3L92514 Corail;* right hip*: acetabular implant, 9998-04-652; taper sleeve adaptor, 9998-00-102; femoral implant, 9998-90-146, femoral stem, 3L92513 Corail.
